# Prospective association of air purifier use during pregnancy with the neurodevelopment of toddlers in the Japan Environment and Children’s Study

**DOI:** 10.1038/s41598-021-98482-y

**Published:** 2021-09-30

**Authors:** Kenta Matsumura, Kei Hamazaki, Akiko Tsuchida, Hidekuni Inadera, The Japan Environment and Children’s Study (JECS) Group, Michihiro Kamijima, Shin Yamazaki, Yukihiro Ohya, Reiko Kishi, Nobuo Yaegashi, Koichi Hashimoto, Chisato Mori, Shuichi Ito, Zentaro Yamagata, Takeo Nakayama, Hiroyasu Iso, Masayuki Shima, Youichi Kurozawa, Narufumi Suganuma, Koichi Kusuhara, Takahiko Katoh

**Affiliations:** 1 Department of Public Health, Faculty of Medicine, University of Toyama, 2630 Sugitani, Toyama, 930-0194 Japan; 2 Toyama Regional Center for JECS, University of Toyama, Toyama, Japan; 3 Department of Public Health, Gunma University Graduate School of Medicine, Gunma, Japan; 4 Nagoya City University, Aichi, Japan; 5 National Institute for Environmental Studies, Tsukuba, Japan; 6 National Center for Child Health and Development, Tokyo, Japan; 7 Hokkaido University, Sapporo, Japan; 8 Tohoku University, Sendai, Japan; 9 Fukushima Medical University, Fukushima, Japan; 10 Chiba University, Chiba, Japan; 11 Yokohama City University, Yokohama, Japan; 12 University of Yamanashi, Chuo, Japan; 13 Kyoto University, Kyoto, Japan; 14 Osaka University, Suita, Japan; 15 Hyogo College of Medicine, Nishinomiya, Japan; 16 Tottori University, Yonago, Japan; 17 Kochi University, Nankoku, Japan; 18 University of Occupational and Environmental Health, Kitakyushu, Japan; 19 Kumamoto University, Kumamoto, Japan

**Keywords:** Neuronal development, Cognitive neuroscience, Public health

## Abstract

We examined the association between maternal air purifier use during pregnancy and neurodevelopmental delay in toddlers by analysing data from 82,457 mother-toddler pairs. Air purifier use was measured using a simple yes/no question. Developmental delays at 1.5, 2.0, 2.5, and 3.0 years were assessed using the Ages and Stages Questionnaire, Third Edition. Generalized additive mixed model analysis with 21 covariates revealed that air purifier use was associated with lower prevalence of developmental delay in all five areas—communication, gross motor, fine motor, problem solving, and personal-social—at all four time points (adjusted risk ratios ranged from 0.827 to 0.927, and only one 95% confidence interval crossed the reference). These findings suggest a negative association between air purifier use during pregnancy and neurodevelopmental delay in toddlers.

Trial registration: UMIN000030786 (15/01/2018).

## Introduction

Accumulating evidence suggests that foetal exposure to air pollution plays a role in the aetiology of neurodevelopmental delay^1–3^. For example, previous studies linked foetal exposure to particulate matter (PM) or polycyclic aromatic hydrocarbons to brain morphology and reduced cognitive function in school-aged children^4^ and decreased IQs at 5 years of age^5^. Although the detailed mechanisms underlying the relationship remain unknown, one possible pathway is via neuroinflammation^6^.

One potential countermeasure against an air pollution-related increase in children’s neurodevelopmental delay would involve the use of air purifiers during pregnancy. Previous studies found that air purifiers can drastically reduce indoor PM1–PM50 concentrations^7,8^ and weaken allergic symptoms by reducing airborne PM2.5 levels^9,10^. Accordingly, children’s neurodevelopmental delay should also be reduced in the offspring of mothers who use an air purifier during pregnancy.

We previously examined this aspect using cohort data from the Japan Environment and Children’s Study (JECS)^11^. Although we did not measure indoor PM concentrations ourselves, we revealed that air purifier use during pregnancy was associated with decreased prevalence of infant neurodevelopmental delay at 6 and 12 months of age, as assessed using the Ages and Stages Questionnaire, Third Edition (ASQ-3). However, given that the human brain undergoes dramatic development both structurally and functionally in early childhood^12^, it is necessary to examine whether this association persists at later ages.

Therefore, to expand on our recent findings^11^, we examined the same topic but focused on data obtained from children between 1.5 and 3 years of age. Fortunately, the JECS data set including this time period has already been released. We hypothesized that a developmental delay would be less common in toddlers whose mother had used an air purifier during pregnancy.

## Methods

### Study design and participants

**Figure 1:**
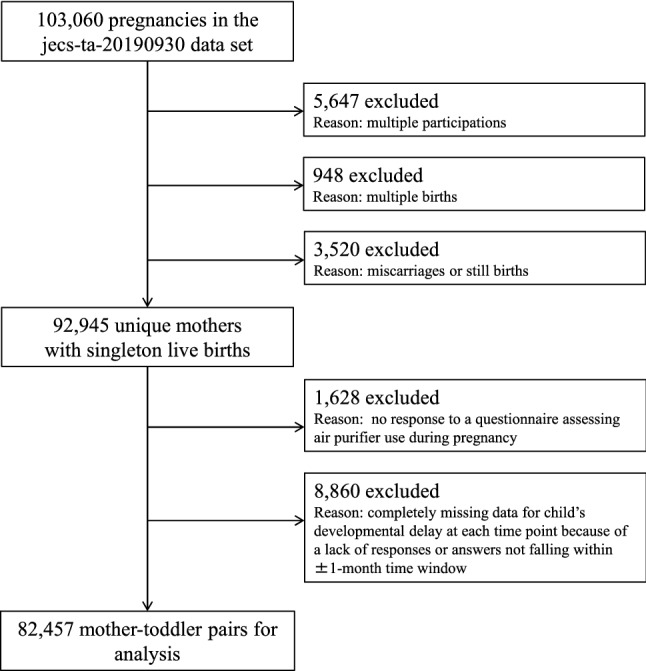
Participants’ flow chart.

The detailed design and baseline characteristics of the JECS have been published elsewhere^13–15^. Briefly, the JECS is a nationwide, government-funded, birth cohort study of various environmental factors and children’s health and development [registered at UMIN.ac.jp as UMIN000030786 (15/01/2018)]. The participants in the JECS were registered via face-to-face recruitment in 15 regional centres throughout Japan between January 2011 and March 2014. The present study analysed the *jecs-ta-20190930* data set, which was released in October 2019. This data set includes data on 103,060 pregnancies up to 3 years postpartum. From this set, we excluded records with inadequate data for analysis, such as those involving multiple participations (the second or third registration of the same mother), multiple births (twins or more), miscarriages/still births, and completely missing data, leaving 82,457 mother-toddler pairs for the final analysis (Fig. 1).

The authors assert that all procedures contributing to this work comply with the ethical standards of the relevant national and institutional committees on research involving human participants and with the Helsinki Declaration of 1975, as revised in 2008. All procedures involving human participants were approved by the Ministry of the Environment’s Institutional Review Board on Epidemiological Studies (no. 100910001) and the Ethics Committee of the University of Toyama (no. R2018023). Written informed consent was obtained from all mother-toddler pairs.

### Measures

#### Exposure

The use of air purifiers during the previous year was assessed as part of a series of questions regarding the dwelling house and environment in the second/third trimester (27.8 ± 6.4 *SD* weeks of gestation) using a simple yes/no question: ‘Have you used an air-cleaning device during the last year?’.

#### Outcomes

Children’s neurodevelopment at 1.5, 2.0, 2.5, and 3.0 years of age was assessed using the ASQ-3TM, an age-specific, structured, parent-completed, child monitoring system^16^. The ASQ-3 is a set of well-validated questionnaires that has been recommended by the United Nations Children’s Fund to verify whether children have normal neurological development^17^. The Japanese version of the ASQ-3 has also been validated^18^ and has already been used in several studies^11,19^. The ASQ-3 assesses the following five areas of development: (a) communication: language skills, such as babbling, vocalizing, listening, and understanding; (b) gross motor: arm, body, and leg movements during movement and play; (c) fine motor: hand and finger movements; (d) problem solving: problem-solving skills, learning, and playing with toys; and (e) personal-social: self-help skills, solitary social play, and play with toys and others. Screen-positive cases for each area are defined as those with scores on or below the cut-off values^18^. Taking early delivery into account, if a parent’s completion dates at 1.5, 2.0, 2.5, and 3.0 years were not within ± 1 month of the estimated delivery date, the data were treated as missing values in accordance with the scoring guidelines.

#### Covariates

We used the same potential confounders that were used in our previous study^11^ together with one new confounder. A priori-selected covariates included maternal age (< 25, 25– < 30, 30– < 35, or ≥ 35 years), body mass index (< 18.5, 18.5– < 25, or ≥ 25 kg/m^2^), parity (primiparous or multiparous), smoking status (never, former, or current), passive smoking status (almost never or 1, 2–3, 4–6, or 7 days/week), alcohol intake (never, former, or current), number of hours spent outdoors (< 1, 1– < 2, 2– < 3, or ≥ 3 h), physical activity (yes or no), folic acid intake^20^ (≤ 151, 152–202, 203–257, 258–337, or ≥ 338 µg) assessed using a food frequency questionnaire^21^, marital status (married, single, or divorced or widowed), highest educational level (≤ 12, > 12– < 16, or ≥ 16 years), employment status (yes or no), and annual household income (< 4, 4– < 6, or ≥ 6 million JPY), type of residence (wooden detached house, steel-frame detached house, wooden multiple dwelling house/apartment, steel-frame multiple dwelling house/apartment, or other), high-rise living^22^ (living ≥ 6th floor or not), number of rooms in the house/apartment (≤ 2, 3, 4, 5, or ≥ 6), living room flooring material (tatami [Japanese straw floor covering], carpet on tatami, wooden flooring/tiles, carpet on wooden flooring/tiles, or other), age of house/apartment building (< 1, 1– < 3, 3– < 5, 5– < 10, 10– < 20, or ≥ 20 years or unknown), house renovation/interior completion after becoming pregnant (yes or no), number of years living in the current place of residence (< 1, 1– < 3, 3– < 5, 5– < 10, 10– < 20, or ≥ 20 years), due date of delivery (months from 1 January 2011), and 19 study areas. These covariates were selected because they are standard variables for socioeconomic status and dwelling environment in Japan as well as because of their possible impact on exposure and/or outcome. For example, mothers with higher socioeconomic status, in terms of higher income and education and living in a new large house, were considered more likely to be able to afford an air purifier and to be concerned with children’s development. Multiparous mothers tend to use air purifiers to keep their house clean and older siblings can affect newborn’s development. In addition, living environment, living place, and the due date of birth can be associated with air pollutant exposure during the potential critical window of foetal development and thus affect both the effectiveness of air purifier use and child development. Potential mediators were not used as covariates. These variables were categorized according to usual medical practice or common practice in Japan and/or by referring to our previous studies^23–25^.

### Statistical analysis

To calculate risk ratios (RRs) and their 95% confidence intervals (CIs), we used separate generalized additive mixed models, with 19 study areas in 15 regional centres set as a random effect, with best-fitted spline as a smoothing function for due date of delivery^26^, with log as a link function, and with binomial and Poisson as distributions for dichotomized and counted outcomes, respectively. Outcome variables were screen-positive cases in the five developmental areas of the ASQ-3: (a) communication, (b) general motor, (c) fine motor, (d) problem solving, and (e) social-personal, as well as (f) the total number of screen-positive cases over the five areas (thus, ranging from 0 to 5). The exposure variable was the yes/no answer to the question regarding air purifier use. Nonuse of air purifiers was used as the reference.

R 4.0.5 mcgv packages including the bam (generalized additive mode for big data) function and SAS 9.4 (SAS Institute Inc., Cary, North Carolina) were used for all statistical analyses.

### Missing data

The effective response rates at 1.5, 2.0, 2.5, and 3.0 years postpartum were 85.9% (n = 79,866), 84.5% (n = 78,535), 82.9% (n = 77,029), and 81.2% (n = 75,494), respectively. The missing data rate was highest for annual household income (n = 5618; 6.8%), followed (in order) by hours spent outdoors (n = 3304; 4.0%), physical activity (n = 2732; 3.3%), and years living in the current place of residence (n = 2570; 3.1%). All other covariates had a missing value rate ≤ 1%.

We conducted imputation using chained equations^27^ to obtain 10 imputed data sets using the MI procedure in SAS. All outcomes and mother’s age, height, and weight as continuous variables and all covariates except for due date of delivery (because of no missing data) as categorical variables were simultaneously imputed. Auxiliary variables that were associated with variables used in the main analysis were introduced to approach the missing at random assumption. For example, we included partner’s highest education level and the number of family members with an income into the imputation model because they effectively predicted income. The ASQ-3 scores at 0.5 and 1.0 years were used to predict the later scores (i.e., 1.5–3.0 years). The estimates from each data set after multiple imputation were combined using Rubin’s rule^28^.

### Sensitivity analysis

Complete case analysis was conducted in addition to the main analysis. We have also checked the assumption of unbiased complete case analysis that missingness depends on the outcome when controlling for all other covariates^29^ (e.g., is income missingness associated with the neurodevelopment score when controlling for other covariates?). We checked the significance of a total of 420 *β*-coefficients (i.e., 21 covariates as used in the main analysis × 5 kinds of outcomes as continuous variables × 4 time points) using linear models.

### Additional analysis

We calculated generalized variance inflation factors to assess multicollinearity.

## Results

The characteristics of the mothers and dwelling environments together with data regarding air purifier use during pregnancy are presented in Table 1. Higher air purifier use was associated with multiple variables; in descending order by strength of relationship, the top five covariates were newer house (Cramer’s *V* = 0.104), higher income (*V* = 0.090), living room flooring materials (*V* = 0.080), multiparous status (*V* = 0.074), and numbers of rooms in the house (*V* = 0.067).

**Table 1:** Characteristics of the participants and dwelling environments.

Variable	Category	Air purifier use				*p*
		Yes		No		
		n	(%)	n	(%)	
Subtotal		41,791	(50.9)	40,362	(49.1)	
**Mothers**						
Age, y	< 25	3563	(8.5)	4359	(10.8)	< 0.001
	25– < 30	12,144	(29.1)	11,054	(27.4)	
	30– < 35	15,439	(37.0)	13,855	(34.3)	
	≥ 35	10,641	(25.5)	11,092	(27.5)	
Body mass index, kg/m^2^	< 18.5	6781	(16.2)	6542	(16.2)	< 0.001
	18.5– < 25	30,977	(74.2)	29,535	(73.2)	
	≥ 25	4010	(9.6)	4261	(10.6)	
Parity	Primiparous	17,184	(41.1)	19,575	(48.5)	< 0.001
	Multiparous	24,595	(58.9)	20,774	(51.5)	
Smoking status	Never	24,464	(59.0)	23,810	(59.4)	< 0.001
	Former	15,588	(37.6)	14,518	(36.2)	
	Current	1448	(3.5)	1733	(4.3)	
Passive smoking status	Almost never	26,658	(64.0)	25,088	(62.3)	< 0.001
	Once a week	5135	(12.3)	4706	(11.7)	
	2–3 times a week	3271	(7.9)	3325	(8.3)	
	4–6 times a week	1890	(4.5)	2043	(5.1)	
	Every day	4734	(11.4)	5102	(12.7)	
Alcohol intake	Never	13,824	(33.4)	13,491	(33.7)	0.001
	Former	26,569	(64.1)	25,423	(63.4)	
	Current	1055	(2.6)	1180	(2.9)	
Number of hours spent outdoors	< 1	7317	(18.2)	7754	(20.0)	< 0.001
	1– < 2	19,466	(48.3)	18,448	(47.7)	
	2– < 3	6586	(16.4)	5879	(15.2)	
	≥ 3	6916	(17.2)	6636	(17.1)	
Physical activity	No	8995	(22.2)	9342	(23.9)	< 0.001
	Yes	31,483	(77.8)	29,746	(76.1)	
Quintile of folic acid intake, μg	≤ 151	7476	(17.9)	8429	(20.9)	< 0.001
	152–202	8273	(19.8)	8100	(20.1)	
	203–257	8764	(21.0)	8115	(20.1)	
	258–337	8569	(20.5)	7922	(19.6)	
	≥ 338	8705	(20.8)	7790	(19.3)	
Marital status	Married	40,217	(96.9)	37,823	(94.6)	< 0.001
	Single	1063	(2.6)	1766	(4.4)	
	Divorced or widowed	222	(0.5)	397	(1.0)	
Highest education level, y	≤ 12	13,350	(32.1)	15,054	(37.4)	< 0.001
	> 12– < 16	18,580	(44.6)	16,342	(40.6)	
	≥ 16	9710	(23.3)	8822	(21.9)	
Employed	No	19,276	(46.4)	17,889	(44.7)	< 0.001
	Yes	22,252	(53.6)	22,141	(55.3)	
Annual household income, million yen	< 4	13,778	(35.2)	16,370	(43.6)	< 0.001
	4– < 6	13,599	(34.7)	11,937	(31.8)	
	≥ 6	11,799	(30.1)	9203	(24.5)	
Due date of delivery (months from 1 Jan 2011), mean ± SD		28.5	± 10.6	27.2	± 10.7	< 0.001
**Dwelling environment**						
Type of residence	Wooden detached house	17,381	(41.8)	16,354	(40.7)	< 0.001
	Steel-frame detached house	2968	(7.1)	2229	(5.6)	
	Wooden multiple dwelling house/apartment	4743	(11.4)	5233	(13.0)	
	Steel-frame multiple dwelling house/apartment	16,110	(38.7)	15,942	(39.7)	
	Other	384	(0.9)	390	(1.0)	
High-rise living	No	39,663	(94.9)	38,704	(95.9)	< 0.001
	Yes	2128	(5.1)	1658	(4.1)	
Number of rooms in house/apartment	≤ 2	6949	(16.7)	8393	(20.9)	< 0.001
	3	13,257	(31.9)	13,185	(32.8)	
	4	8591	(20.7)	7055	(17.6)	
	5	6731	(16.2)	5598	(13.9)	
	≥ 6	6067	(14.6)	5942	(14.8)	
Living room flooring materials	Tatami (Japanese straw floor covering)	3880	(9.3)	5365	(13.3)	< 0.001
	Carpet on tatami	3206	(7.7)	4012	(10.0)	
	Wooden flooring/tiles	15,663	(37.6)	13,576	(33.8)	
	Carpet on wooden flooring/tiles	18,127	(43.5)	16,589	(41.2)	
	Other	794	(1.9)	688	(1.7)	
Age of house/apartment building, y	< 1	2732	(6.6)	1959	(4.9)	< 0.001
	1– < 3	5470	(13.2)	3826	(9.5)	
	3– < 5	4373	(10.5)	3447	(8.6)	
	5– < 10	6846	(16.5)	5858	(14.6)	
	10– < 20	9469	(22.8)	9613	(24.0)	
	≥ 20	9438	(22.7)	11,013	(27.5)	
	Unknown	3235	(7.8)	4404	(11.0)	
House renovation/interior finished after getting pregnant	No	40,135	(96.5)	38,986	(97.1)	< 0.001
	Yes	1446	(3.5)	1161	(2.9)	
Number of years living in current place of residence	< 1	2754	(6.8)	2686	(6.9)	< 0.001
	1– < 3	17,781	(43.8)	16,705	(42.7)	
	3– < 5	9588	(23.6)	8452	(21.6)	
	5– < 10	7164	(17.6)	7064	(18.1)	
	10– < 20	1937	(4.8)	2308	(5.9)	
	≥ 20	1410	(3.5)	1888	(4.8)	

Cases and prevalence of developmental delay are presented in Table 2. All prevalences were lower in the air purifier use group than in the air purifier nonuse group.

**Table 2:** Cases and prevalence of developmental delay at four time points in various areas of the ASQ-3 and the total number of cases according to use or nonuse of air purifiers.

	Communication		Gross motor		Fine motor		Problem solving		Personal-social		Total number	
	n	(%)	n	(%)	n	(%)	n	(%)	n	(%)	M	S.E.M
**1.5 years**												
AP use ^a^	954	(2.3)	1889	(4.5)	1622	(3.9)	1460	(3.5)	963	(2.3)	0.16	± 0.0029
AP nonuse ^b^	1061	(2.6)	2201	(5.4)	1848	(4.6)	1797	(4.4)	1124	(2.8)	0.20	± 0.0032
**2.0 years**												
AP use ^a^	1583	(3.8)	2390	(5.7)	879	(2.1)	1634	(3.9)	1035	(2.5)	0.18	± 0.0031
AP nonuse ^b^	1746	(4.3)	2632	(6.5)	1013	(2.5)	1996	(4.9)	1309	(3.2)	0.21	± 0.0033
**2.5 years**												
AP use ^a^	1842	(4.4)	1766	(4.2)	2138	(5.1)	2172	(5.2)	1375	(3.3)	0.22	± 0.0035
AP nonuse ^b^	2060	(5.1)	1923	(4.8)	2447	(6.0)	2435	(6.0)	1529	(3.8)	0.26	± 0.0039
**3.0 years**												
AP use ^a^	1587	(3.8)	1760	(4.2)	2880	(6.9)	2765	(6.6)	1342	(3.2)	0.25	± 0.0038
AP nonuse ^b^	1712	(4.2)	1980	(4.9)	3243	(8.0)	3032	(7.5)	1491	(3.7)	0.28	± 0.0041

^a^n = 41,951. ^b^n = 40,506.

Based on imputed data for the 82,457 mother-toddler pairs in this study.

ASQ-3 = the Ages and Stages Questionnaire, Third Edition, AP = air purifier.

**Figure 2:**
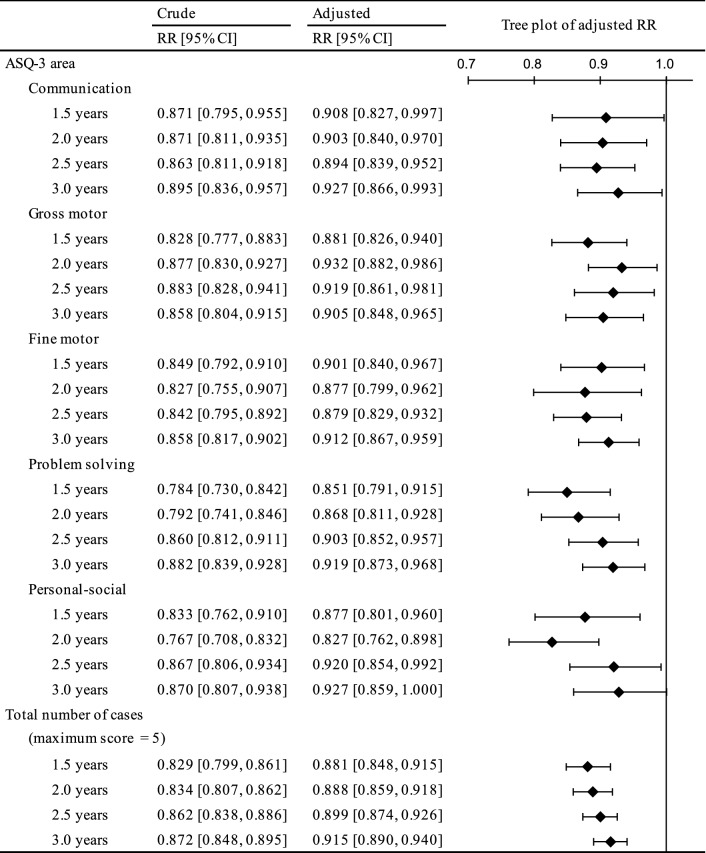
Tree plot of adjusted risk ratios (RRs) and 95% confidence intervals (CIs) for cases of developmental delay at four time points in various areas of the Ages and Stages Questionnaire, Third Edition, and the total number of cases with air purifier use (reference = nonuse of air purifiers). Based on imputed data for the 82,457 mother-toddler pairs in this study. Adjusted for maternal age, body mass index, parity, smoking status, passive smoking status, alcohol intake, number of hours spent outdoors, physical activity, folic acid intake, marital status, highest educational level, employment status, annual household income, type of residence, high-rise living, number of rooms in house/apartment, living room flooring material, age of house/apartment building, house renovation/interior completion after becoming pregnant, number of years living in current place of residence, and due date of delivery, with the 19 regional areas set as a random effect.

Crude and adjusted RRs are presented in Fig. 2. All RRs were below the reference (= 1). They ranged from 0.767 to 0.927 and all but one 95% CI did not cross 1.

The results of complete case analysis were largely unchanged (Supplementary Fig. 1). In addition, analysis checking the unbiased assumption of complete case analysis detected only 11 significant *β*-coefficients out of a total of 420 ASQ-3 scores. Given that 21 significant *β*-coefficients were expected (i.e., *α* = 0.05), the result suggests that the current complete case analysis yielded unbiased results.

Multicollinearity was not detected among any covariate; that is, all generalized variance inflation factors were below 2.73.

## Discussion

As an extension of our previous study^11^, we used recently fixed JECS data and examined the prospective relationship between the use of air purifiers during pregnancy and neurodevelopmental delay in toddlers up to 3 years of age. Our analysis revealed fewer cases of neurodevelopmental delay in toddlers who were born to mothers who had used an air purifier during pregnancy compared with children of mothers who had not used a purifier. These findings suggest that air purifier use during pregnancy is associated with lower prevalence of neurodevelopmental delay in offspring, despite the developmental outcomes being set to 1.5 to 3 years of age.

Previous studies examined school-aged children^4^ and 5-year-old children^5^ at a single time point. In contrast, the current series of studies examined children up to 3 years of age at fixed intervals of 6 months. Taken together with our previous findings that focused on a neurodevelopmental delay up to 1 year of age^11^, we can conclude that the trajectory of the difference between the use or nonuse of air purifiers appeared as early as at 6 months of age, with the difference further increasing from 1 to 1.5 years of age and remaining the same until at least 3 years of age. Although the difference tended to decrease at 3 years of age in terms of the total number of cases, the tendency was not as clear. In addition, taken together with the previous detection of differences at later ages^4,5^, the possibility that the difference detected in our data at 3 years of age disappears immediately is likely to be very low.

Although the present study revealed that the maternal use of air purifiers was related to the development of their offspring until at least 3 years of age, this association is not causal but prospective. This means that there is no guarantee that the use of air purifiers during pregnancy would protect against neurodevelopmental delay in offspring. Furthermore, it remains unknown which property of air purifier use would mitigate neurodevelopmental delay, although we considered indoor PM to be one factor and used multiple possible confounders that affect exposure and/or outcome to adjust the model. This is because, firstly, we did not measure actual indoor PM levels. Secondly, we did not measure the ability of the air purifiers to reduce the exposure of mothers to such pollutants. Thus, future studies should examine the mechanism underlying the association. In addition to the underlying mechanism, a randomized controlled trial should be conducted to examine causality. Given that not all medications have a clear mechanism of action, a preventive method itself should be developed. Taken together, while the association found in the present study is itself clear, the meaning of this association and how useful it is for preventing neurodevelopmental delay need to be examined further.

The strengths of the study are as follows. First, our sample size was large. We analysed over 82,000 mother-toddler pairs. Second, our sample was recruited from 15 regional centres throughout Japan. In addition, our sample was recruited within the past 10 years. Thus, our sample is plausibly representative of recent Japanese mother-toddler pairs. Third, the dropout rate was relatively low, at about 18.8%, despite the 3-year follow-up. In addition, we imputed data. Thus, any selection bias was likely to be small. Finally, we introduced multiple potential confounders, including not only demographic, socioeconomic, and dwelling environmental variables, but also time and location variables, that can impact air purifier use and neurodevelopment through a potential critical window of pollutant exposure and foetal development. This means that the unpredictable effects of unmeasured confounders should be minimal.

A number of limitations also need to be considered. First, we measured neurodevelopmental delay using the ASQ-3. Although the ASQ-3 is a well-validated questionnaire, it is neither a diagnostic nor objective measure. Thus, although acceptable, the objectivity of the measures is not as high as it could be. Second, we measured the use of air purifiers via a very simple yes/no question. Thus, further analyses, such as one involving a dose–response study using the duration and/or frequency of air purifier use and the numbers owned while controlling for important confounders, such as filter type and household location^8^ as well as air pollutant levels and meteorological data^30^, that can more directly impact air purifier use and foetal neurodevelopment, were impossible. Finally, because we did not measure indoor PM concentrations, mediation analysis using the PM concentration was impossible.

In conclusion, we found a negative prospective association between air purifier use during pregnancy and neurodevelopmental delay in offspring up to 3 years of age.

## Supplementary Information

Supplementary Information.

## Data Availability

Data are unsuitable for public deposition due to ethical restrictions and the legal framework of Japan. It is prohibited by the Act on the Protection of Personal Information (Act No. 57 of 30 May 2003, amendment on 9 September 2015) to publicly deposit data containing personal information. Ethical Guidelines for Medical and Health Research Involving Human Subjects enforced by the Japan Ministry of Education, Culture, Sports, Science and Technology and the Ministry of Health, Labour and Welfare also restrict the open sharing of epidemiologic data. All inquiries about access to data should be sent to: jecs-en@nies.go.jp. The person responsible for handling enquiries sent to this e-mail address is Dr Shoji F. Nakayama, JECS Programme Office, National Institute for Environmental Studies.
