# Supplementary material for: Prospective association of air purifier use during pregnancy with the neurodevelopment of toddlers in the Japan Environment and Children’s Study
Source: Sci Rep. 2021 Sep 30;11:19454. doi: 10.1038/s41598-021-98482-y (PMC8484572; doi:10.1038/s41598-021-98482-y)
Supplement: Supplementary file 1 — Supplementary Information. [file 41598_2021_98482_MOESM1_ESM.docx]

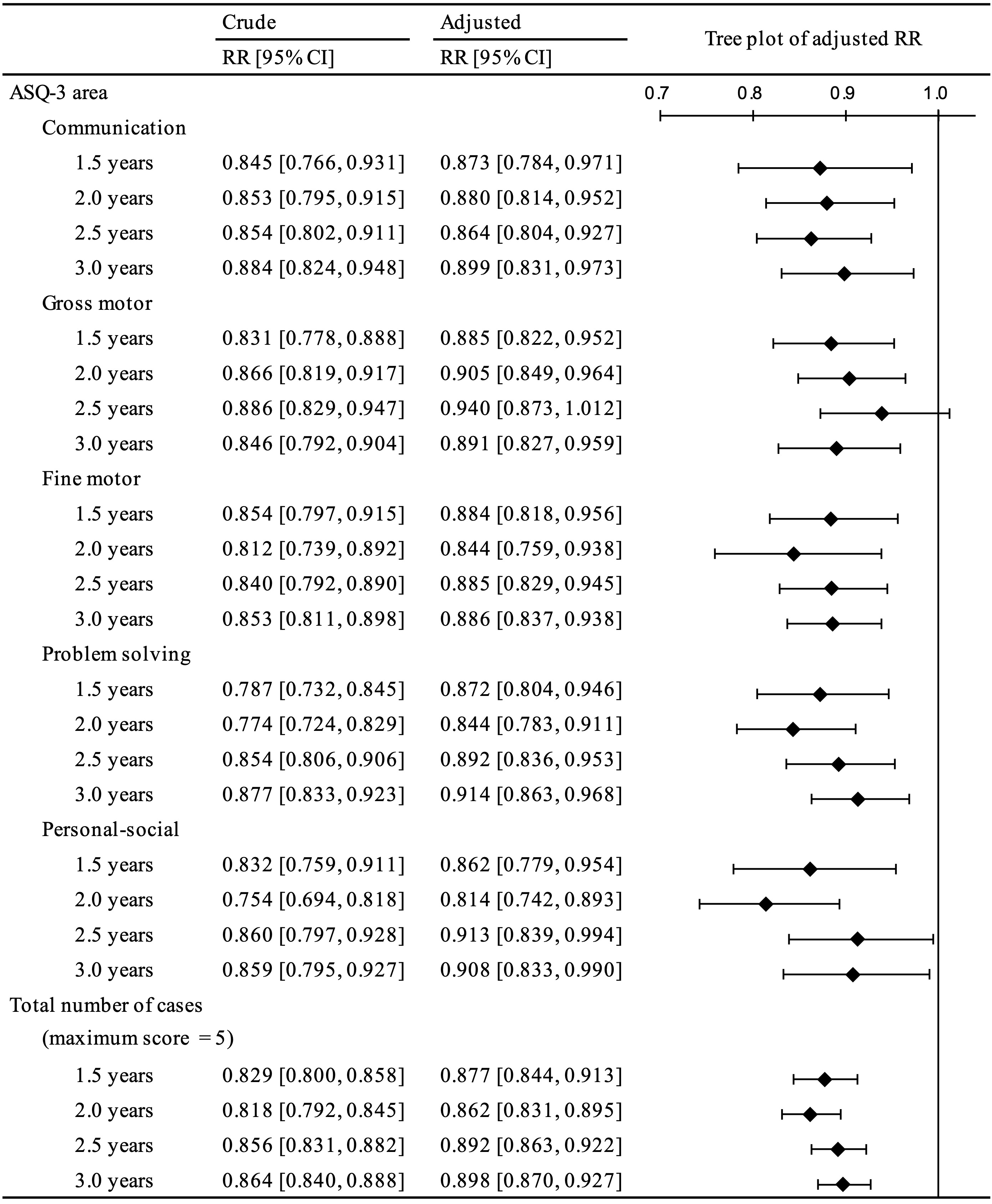


**Supplementary Figure 1.** Tree plot of adjusted risk ratios (RRs) and 95% confidence intervals (CIs) for cases of developmental delay at four time points in the various subsections of the Ages and Stages Questionnaire, Third Edition, and the total number of cases with air purifier use (reference = nonuse of air purifiers) calculated from complete case analysis.

Adjusted for maternal age, body mass index, parity, smoking status, passive smoking status, alcohol intake, number of hours spent outdoors, physical activity, folic acid intake, marital status, highest educational level, employment status, annual household income, type of residence, high-rise living, number of rooms in house/apartment, living room flooring material, age of house/apartment building, house renovation/interior completion after becoming pregnant, number of years living in current place of residence, and due date of delivery, with the 19 regional areas set as a random effect.
